# Prolonged Opioid Use Following Distal Radius Fracture Fixation: Who Is at Risk and What are the Consequences?

**DOI:** 10.1016/j.jhsg.2023.03.003

**Published:** 2023-03-31

**Authors:** William Runge, Andrew M. Gabig, Anthony Karzon, Nina Suh, Eric R. Wagner, Michael B. Gottschalk

**Affiliations:** ∗ Investigation performed at the Emory Clinic, Atlanta, GA

**Keywords:** Consumption, Distal radius, Fixation, Opioid, Risk factors

## Abstract

**Purpose:**

Opioid pain medication is most commonly prescribed after distal radius fracture fixation, and there is high variability in the quantity and duration prescribed. Comorbidities, including substance use and depression, have been associated with higher consumption habits, and increased sizes of postoperative opioid prescriptions have been previously linked to an increasing risk of chronic opioid use and opioid use disorder. The purpose of this study was to investigate opioid prescription patterns after distal radius fracture fixation and identify patient-specific risk factors associated with increased opioid prescription refills.

**Methods:**

A retrospective review of 34,629 opioid-naïve patients was conducted using the IBM MarketScan database. The database was queried to identify patient records from January 2009 to December 2017. Demographic, comorbidity, complication data, and prescription pharmacy claims were analyzed. Patients were sorted according to the duration of postoperative prescription refills of opioid pain medication.

**Results:**

Seventy-three percent of the patients required no additional refills outside the perioperative window. Twenty percent required additional refill prescriptions, and 6.4% of patients continued to fill the opioid medication beyond 6 months after surgery. Multiple factors increased the risk of increased opioid use, including medical and surgical complications, substance use, diabetes, cardiovascular disease, and obesity. Patients with a longer duration of opioid use after surgery had higher rates of medical and surgical complications. Perioperative prescription quantities were 62.9, 78.6, and 83.3 tablets for no refill, refill (<6M), and prolonged-use groups (>6M), respectively.

**Conclusions:**

Patients who underwent distal radius fracture fixation were at greater odds for prolonged opioid use after surgery in the presence of comorbid cardiovascular, renal, metabolic, and mental health illnesses and postoperative medical and surgical complications. A greater understanding of patient-specific factors for prolonged opioid consumption after distal radius fracture fixation can help providers identify at-risk patients who would benefit from a tailored approach to counseling and multimodal pain management. Patients should be educated on these risks associated with their surgery and be provided with alternative medical options and health care resources to optimize pain control and reduce their need for opioid medication as their primary tool for pain relief.

**Type of study/level of evidence:**

Therapeutic III.

Pain control after orthopedic interventions has become a significant focus of research in recent years.^1^^,^^2^ Distal radius fractures (DRFs) are among the most common bony injuries treated by hand surgeons, and the incidence of operative fixation is increasing in incidence.^3^^,^^4^ Opioid pain medication is most commonly prescribed after surgery, and there is high variability in the quantity and duration prescribed after surgical fixation.^5^ Since 2000, a steep increase in the availability, prescription frequency, and consumption of opioid medication has played a significant factor in the opioid overdose epidemic.^6^ Since the Joint Commission’s labeling of pain as the fifth vital sign, the US population consumes 80% of the global opioid supply, and there has been a significant increase in the prevalence of opioid use disorder in the United States.^7^

Among physicians in the United States, hand surgeons are the third highest prescribers of opioid medication, and surgeons should understand the negative consequences opioid pain medication may have on both patients’ clinical outcome and life after their injury.^8^ A significant discrepancy has been identified between the prescription and consumption habits of opioid pain medication in many patients undergoing hand surgery.^2^^,^^9^ Higher initial prescriptions of opioid pain medication have a strong correlation with a patient’s risk of opioid dependence and misuse.^10^^,^^11^ Further evidence suggests that certain comorbidities are associated with prolonged opioid use, and prolonged use of opioid medication maybe less related to surgical pain than it is to addressable patient factors.^7^^,^^12^^,^^13^ Previous analyses of prescribing patterns after upper extremity and hand surgery identified elective surgery, younger age, female patients, and lower incomes as risk factors associated with a longer duration of opioid consumption.^14^ Potential causal associations between a genetic liability for prescription opioid use and major depressive disorder and anxiety and stress-related disorders has been suggested^15^; however, there is a paucity of research identifying the impact of these comorbid conditions on the long-term use of opioid medication after surgical interventions.

Multiple studies have also demonstrated correlations between the preoperative use of opioids and increased rates of complications, worse patient outcomes, and increased health care utilization.^10^^,^^16^ Although the link between opioid use and increased complications is actively under investigation, opioids have been shown to negatively affect wound healing and the inflammatory process.^17^ Identifying patients who are more likely to have a higher consumption of opioid medications is vitally important for surgeons because these are at risk for suboptimal outcomes and opioid dependence.

The purpose of this study was to investigate opioid prescription patterns after distal radius fracture fixation and identify patient-specific risk factors for increased postoperative opioid consumption. We hypothesized that factors such as substance abuse, depression, and medical comorbidities will increase the risk of additional opioid use. In addition, we hypothesized that higher perioperative prescription quantity and certain perioperative complications will increase the risk of prolonged opioid use.

## Materials and Methods

### Data acquisition and patient population

Data were collected using the IBM Watson Health MarketScan Commercial Claims and Encounters, Medicare Supplemental, and Coordination of Benefits Databases. The MarketScan database is a nationwide insurance database that includes de-identified longitudinal health and demographic information related to millions of privately and publicly insured patients in the United States. Institutional review board approval was not required for this study.

The database was searched for patients undergoing outpatient open reduction internal fixation of a distal radius fracture between January 2009 and December 2017 using the Current Procedural Terminology codes 25607 (open treatment of extra-articular distal radius fracture), 25608 (open treatment of intra-articular distal radius fracture with internal fixation of two fragments), and 25609 (open treatment of intra-articular distal radius fracture with internal fixation of three or more fragments). This study period was limited to the data-years made available by MarketScan to our institution at the time of the analysis. Patients aged 18 years or older at the time of the surgery and had continuous enrollment in the database for at least 6 months before surgery and 12 months after surgery were included. Outpatient pharmacy claims were analyzed for opioid prescriptions using National Drug Codes. Oral morphine equivalents (OME) were calculated by multiplying the prescription quantity by a conversion factor specific to each opioid medication.^18^

The study was broken down into four separate periods: preoperative (6 months to 2 weeks preoperative), perioperative (2 weeks preoperative to 2 weeks postoperative), postoperative (2 weeks to 6 months postoperative), and prolonged use (>6 months postoperative). Any opioid prescriptions filled within the perioperative window were cumulatively combined to calculate the perioperative prescription quantities, and we assumed that the intended use of this prescription would be for postoperative pain management. Patients who were not prescribed an opioid medication in the perioperative window were excluded.

Only opioid-naïve patients—defined as zero opioid prescription claims within the preoperative period—were included in the analysis. Prescriptions for opioid medication within the perioperative period were aggregated. Patients were separated into one of the three groups based off their postoperative opioid prescription claims: “No Refill” includes those with no additional opioid prescription refills outside the perioperative window, “Refill (<6M)” includes any additional opioid prescription refill within the postoperative period, and “Prolonged Use (>6M)” includes any patients with opioid refills within both the postoperative and prolonged use periods indicated their continued use of opioid medication for greater than 6 months after DRF fixation.

### Identification of patient risk factors and complications

Baseline patient data were collected based on International Classification of Diseases codes and is shown in Table 1. Complications were similarly identified and including surgical complications of acute infection, hardware and wound complications, and medical complications including myocardial infarction, pneumonia, sepsis, stroke, thromboembolic events, emergency department encounter, and hospital readmission.

**Table 1:** Demographics and Comorbidities of Postoperative Opioid Use Groups after Distal Radius Fracture Fixation

Characteristics	Postoperative Opioid Use Groups			P Value∗
	No Refill	Refill (<6M)	Prolonged Use (>6M)	
Total, *n* (%)	25,427 (73.4)	6,992 (20.2)	2,210 (6.4)	
Age Group				
18–49	7,869 (30.9)	2,248 (32.2)	672 (30.4)	**<.001**
50–64	12,647 (49.7)	3,584 (51.3)	1,108 (50.1)	
65+	4,911 (19.3)	1,160 (16.6)	430 (19.5)	
Sex				
Men	6,547 (25.7)	1,986 (28.4)	558 (25.2)	**<.001**
Women	18,880 (74.3)	5,006 (71.6)	1,652 (74.8)	
Comorbidities				
Obesity	931 (3.7)	344 (4.9)	120 (5.4)	**<.001**
Renal Disease	341 (1.3)	97 (1.4)	56 (2.5)	**<.001**
Alcohol Abuse	231 (0.9)	114 (1.6)	52 (2.4)	**<.001**
Tobacco Use	1,064 (4.2)	483 (6.9)	175 (7.9)	**<.001**
Hypertension	6,250 (24.6)	1,990 (28.5)	681 (30.8)	**<.001**
Hyperlipidemia	5,627 (22.1)	1,560 (22.3)	574 (26.0)	**<.001**
Coronary Artery Disease	910 (3.6)	272 (3.9)	131 (5.9)	**<.001**
Congestive Heart Failure	275 (1.1)	79 (1.1)	49 (2.2)	**<.001**
Diabetes	1,811 (7.1)	634 (9.1)	233 (10.5)	**<.001**
Rheumatic Disease	252 (1.0)	88 (1.3)	25 (1.1)	.142
Depression	1,784 (7.0)	634 (9.1)	295 (13.3)	**<.001**
Anxiety	889 (3.5)	297 (4.2)	116 (5.2)	**<.001**

∗ *P* value denotes any statistically significant differences between any group: presented as *n* (%). Significant values are highlighted in bold.

### Statistical analysis

All statistical analyzes were performed using SPSS software. A *P* value of <.05 was considered significant. One-way ANOVA with post-hoc comparison and binomial logistic regressions were carried out for the statistical analysis of independent risk factors associated with additional opioid refills while controlling for the baseline demographic and comorbidity data. Odds ratios with 95% confidence intervals were calculated for variables with *P* values of <.05. Comparisons were made between groups receiving no refills, postoperative period refills, and prolonged use period refills.

## Results

A total of 34,629 opioid-naïve patients were identified as meeting the inclusion criteria from 2009 to 2017. Of these, 25,427 (73.4%) required no refills, 6,992 (20.2%) required additional prescriptions in the postoperative period, and 2,210 (6.4%) met the criteria for prolonged use. The average age was 53.98 (± 14.8) years, and 73.2% of the patients were women.

Chi-Square analyses revealed significant baseline differences in the demographic and comorbidity data between the three subgroups. Patients older than the age of 65 years and female biological sex had significantly lower rates of prolonged opioid use after DRF fixation (*P* < .001). Significant differences existed in the presence of comorbidities, including obesity, substance use, diabetes, congestive heart failure, depression, and anxiety. The prevalence of each of these was consistently highest in the prolonged-use group (Table 1).

Odds ratios (ORs) were calculated comparing comorbidity and complication presence between “No Refill” and “Refill (<6M)” or “Prolonged Use (>6M)” groups (Table 2). Factors associated with decreased risk included age >65 years (OR, .74), female sex (OR, .91), and hyperlipidemia (OR, .92). Predictive comorbid conditions that may increase opioid use risk include tobacco use (OR, 1.63), alcohol abuse (OR, 1.44), diabetes (OR, 1.24), hypertension (OR, 1.26), depression (OR, 1.22), and obesity (OR, 1.22). Postoperative complications after DRF fixation are not able to be predictive of future use but are otherwise associated with significantly increased risk of opioid use. These included hardware complications (OR, 5.77), hospital readmission (OR, 2.41), acute postoperative infection (OR, 1.97), emergency department (ED) visit (OR, 1.75), and wound complications (OR, 1.74).

**Table 2:** Adjusted Odds of Risk Factors for Postoperative Opioid Use After Distal Radius Fracture Fixation

Risk Factor	Postoperative Opioid Use Groups			
	Refill (<6M)		Prolonged Use (>6M)	
	Odds Ratio∗	*P* Value	Odds Ratio∗	*P* Value
Age Group				
18–49	[Reference]	-	[Reference]	-
50–64	0.98 (0.92–1.04)	.516	0.95 (0.85–1.05)	.310
65+	**0.74 (0.68–0.81)**	**< .001**	**0.77 (0.66–0.89)**	**< .001**
Sex				
Men	[Reference]	-	[Reference]	-
Women	**0.91 (0.85–0.97)**	**.003**	1.05 (0.94–1.17)	.381
Comorbidities				
Obesity	**1.22 (1.07–1.39)**	**.003**	**1.24 (1.01–1.52)**	**.039**
Renal Disease	0.91 (0.72–1.16)	.447	**1.41 (1.04–1.91)**	**.026**
Alcohol Abuse	**1.44 (1.14–1.82)**	**.002**	**1.83 (1.33–2.52)**	**< .001**
Tobacco Use	**1.63 (1.45–1.82)**	**< .001**	**1.78 (1.50–2.11)**	**< .001**
Hypertension	**1.26 (1.17–1.34)**	**< .001**	**1.20 (1.07–1.34)**	**.001**
Hyperlipidemia	**0.92 (0.85–0.98)**	**.016**	1.03 (0.92–1.16)	.569
Coronary Artery Disease	1.00 (0.86–1.16)	.966	**1.33 (1.08–1.64)**	**.007**
Congestive Heart Failure	0.98 (0.75–1.27)	.849	**1.47 (1.05–2.05)**	**.024**
Diabetes	**1.24 (1.11–1.37)**	**< .001**	**1.31 (1.12–1.53)**	**.001**
Rheumatic Disease	1.25 (0.98–1.61)	.078	1.04 (0.69–1.59)	.840
Depression	**1.22 (1.11–1.35)**	**< .001**	**1.75 (1.52–2.01)**	**< .001**
Anxiety	1.10 (0.96–1.26)	.188	1.14 (0.92–1.41)	.228
Surgical Complication				
Acute Infection	**1.59 (1.16–2.16)**	**.003**	**1.97 (1.30–2.99)**	**.001**
Hardware Complication	**5.32 (3.73–7.61)**	**< .001**	**5.77 (3.58–9.29)**	**< .001**
Wound Complication	**1.73 (1.26–2.39)**	**.001**	**1.74 (1.09–2.77)**	**.021**
Medical Complication				
Myocardial Infarction	0.57 (0.23–1.38)	.210	0.50 (0.14–1.75)	.280
Pneumonia	1.07 (0.59–1.91)	.832	1.01 (0.44–2.31)	.988
Sepsis	1.61 (0.72–3.62)	.249	0.97 (0.29–3.21)	.963
Stroke	1.22 (0.90–1.66)	.194	**1.57 (1.05–2.34)**	**.028**
Thromboembolic Event	1.12 (0.75–1.65)	.586	1.03 (0.57–1.84)	.933
ED Visit	**1.61 (1.47–1.76)**	**< .001**	**1.75 (1.52–2.00)**	**< .001**
Readmission	**1.92 (1.66–2.23)**	**< .001**	**2.41 (1.97–2.96)**	**< .001**

ED, emergency department.

∗ Compared with patients without any opioid refills. Presented as adjusted odds ratio (95% confidence interval). Significant values are highlighted in bold.

Medical and surgical complications were significantly different between the groups (Table 3). The presence of surgical complications, including acute infection, hardware complications, and wound complications, was significantly higher in the prolonged-use patient (*P* < .001). Sepsis, stroke, thromboembolic event, ED utilization, and hospital readmission were significantly increased in the prolonged-use group (*p* < .001).

**Table 3:** 90-Day Complications Between Opioid Use Groups After Distal Radius Fracture Fixation

Complication	Postoperative Opioid Use Groups			P Value∗
	No Refill	Refill (<6M)	Prolonged Use (>6M)	
Surgical				
Acute Infection	122 (0.5)	71 (1.0)	31 (1.4)	**<.001**
Hardware Complication	52 (0.2)	80 (1.1)	28 (1.3)	**<.001**
Wound Complication	102 (0.4)	68 (1.0)	24 (1.1)	**<.001**
Medical				
Myocardial Infarction	20 (0.1)	7 (0.1)	3 (0.1)	.622
Pneumonia	38 (0.1)	20 (0.3)	8 (0.4)	**.011**
Sepsis	12 (0.0)	15 (0.2)	4 (0.2)	**<.001**
Stroke	160 (0.6)	63 (0.9)	33 (1.5)	**<.001**
Thromboembolic Event	90 (0.4)	39 (0.6)	15 (0.7)	**.009**
ED Visit	1,966 (7.7)	935 (13.4)	353 (16.0)	**<.001**
Readmission	555 (2.2)	374 (5.3)	167 (7.6)	**<.001**

ED, emergency department.

∗ *P* value denotes any statistically significant differences between any group. Presented as *n* (%).

The average numbers of opioid pills prescribed in the perioperative window were 62.9, 78.6, and 83.3 for the No Refill, Refill (<6M), and Prolonged Use (>6M), respectively (Table 4). The total perioperative OME’s prescribed were 462.0, 575.7, and 609.0 for the groups, respectively. There was a significant difference between the quantity and OME prescribed and between the Refill (<6M) and Prolonged Use (>6M) groups compared with the No Refill groups (*P* < .001). Table 5 describes the trends in perioperative opioids prescribed annually from 2009 to 2017. A weak negative correlation existed between perioperative OME’s prescribed and the year of prescription (r = −.053, *P* < .001). To compare whether there was a change in opioid-prescribing patterns toward the end of the study period compared with the beginning, an analysis between the years 2009 to 2013 and 2014 to 2017 was conducted. Although there were significant decreases in the quantity of opioids prescribed (73 vs 69, *P* < .001), when looking at OMEs prescribed between the two periods, there is a paradoxical increase (831 vs 1089, *P* < .001).

**Table 4:** Opioid Prescriptions in the Perioperative Window by Opioid Use Groups after Distal Radius Fracture Fixation

	No Refill	Refill (<6M)	Prolonged Use (>6M)
Quantity Prescribed∗	62.9 (62.5–63.3)	78.6 (77.7–79.6)	83.3 (81.5–85.0)
Average Difference	0	15.7 (15.2–16.3)	20.4 (18.6–21.7)
*P* Value†	-	**<.001**	**<.001**
OME Prescribed∗	462.0 (458.8–465.2)	575.7 (568.6–582.8)	609.0 (595.7–622.2)
Average Difference	0	113.7 (109.8–117.6)	147.0 (136.9–257.0)
*P* Value†	-	**<.001**	**<.001**

OME, oral morphine equivalents.

∗ Presented as mean (95% confidence interval). Includes all opioid prescriptions in 2-week preoperative and 2-week postoperative.

† *P* value when compared with the no refill group. Significant values are highlighted in bold.

**Table 5:** Yearly Trends in Opioid Perioperative Prescription Quantity and Morphine Equivalents Prescribed

Year	No Refill		Refill (<6M)		Prolonged Use (>6M)	
	MME	QTY	MME	QTY	MME	QTY
2009	679	67	767	84	1461	90
2010	785	68	928	87	1052	95
2011	623	68	793	86	888	96
2012	779	68	872	88	1021	95
2013	1057	67	1027	86	1045	91
2014	916	66	1156	89	1324	98
2015	1434	67	934	86	1330	95
2016	940	64	1034	85	1172	93
2017	1010	58	1150	80	1073	86

Perioperative quantities include 2-weeks preoperative and 2 weeks postoperative.

orphine milligram equivalents; QTY, quantity.

## Discussion

This study sought to identify the comorbidities and risk factors associated with increased use of opioid pain medication after DRF fixation, and the results demonstrate many significant factors that correlate with higher opioid consumption habits. Research continues to discover evidence on the significant negative effects of opioid pain medication, and the need to identify patients who may be at a higher risk for prolonged consumption of opioid medication is more important now than ever. The comorbid conditions associated with a higher risk for opioid consumption—namely, substance abuse, diabetes, depression, obesity, and cardiovascular disease—have been supported in previous studies.^10^^,^^15^^,^^19^ These conditions are common in the population, and the presence of multiple comorbid risk factors may further predispose these patients to prolonged use. Patients have also reported lower levels of pain and consumption of pain medication after DRF injuries compared with other common orthopedic fractures, making DRF procedures a feasible target for further reduced opioid prescription habits.^20^, ^21^, ^22^

Before addressing the results of the study, the authors want to first address the large perioperative prescription quantities reported here. The reported data considered multiple prescriptions that were filled by any one patient within the two weeks before and 2 weeks after the date of surgery. These quantities are unable to account for multiple prescribing physicians or the reason for prescription; however, the results quantify the access patients had to opioid medication in the perioperative window. Although prescribing trends have changed drastically because physicians have better understood the impact opioid medication can have on patients,^22^ patients were being prescribed large quantities and multiple prescriptions in the perioperative window. Resources such as the Prescription Drug Monitoring Program^23^ monitor the prescriptions of controlled substances in a state and allow the physicians to audit the prescription history before dispensing any new medication. This database may not have been as easily accessed during the study period, and referencing the Prescription Drug Monitoring Program has been mandated in many states as a part of modern prescribing habits.^24^ In addition, the data describe a decrease in quantity but a paradoxical increase in OME when comparing 2009 to 2013 and 2014 to 2017. Given the lack of granularity in the data, we cannot make any definitive assumptions; nevertheless, a lower quantity of more potent opioids could partially explain this trend.

We identified significant differences between perioperative prescription trends and the length of opioid consumption, a finding that has been consistently reported in previous studies.^8^^,^^10^^,^^11^ Prescribing higher quantities may be intended to increase patient satisfaction and decrease postoperative complaints; however, these results demonstrate that access to these drugs may not have a benign effect. Multiple studies have attempted to investigate the opioid-prescribing patterns after orthopedic intervention and suggested optimal prescribing recommendations.^2^^,^^9^^,^^10^^,^^25^^,^^26^ Kim et al^9^ analyzed a prospective cohort of 1,416 patients and found the consumption of prescribed pain pills in wrist surgery to be just 27%. Dwyer et al^2^ further reported an average consumption of 16 opioid pills for volar distal radius plate fixation and recommends a prescription regimen of 20 to 30 pills after surgery. These findings support the notion that surgeons have tendencies to prescribe a surplus of pain medication; nevertheless, there is no clear consensus on the optimal prescribing quantity. This surplus may allow an otherwise high-risk patient an opportunity for chronic opioid dependence. Addressing this issue, Jaimeson et al^25^ proposed a standardized postoperative pain plan involving provider and patient education, multimodal analgesia, and a patient-specific opioid calculator that resulted in reduced quantities prescribed, consumed, and wasted. Stepan et al^26^ further demonstrated the benefit of provider education, resulting in 52.3% reduction in prescribed opioids after upper-extremity surgery. Education for prescribers and patients with deliberate prescribing protocols for postsurgical pain management is the first step for surgeons to reduce the risk of prolonged opioid use.

A finding worth describing is the association of prolonged opioid use and complication frequency. An increased consumption of opioids would be expected for patients experiencing significant surgical or medical complications. Any further operative intervention or hospitalization may warrant additional prescriptions. The correlation between increased preoperative opioid use and worsened outcomes has also been previously published.^16^^,^^27^ Blevins Peratikos et al^27^ published results indicating an increased risk of complications and revisions in patients with higher preoperative opioid consumption. Their results of over 34,000 patients indicated a dose-dependent risk relationship with preoperative OME and length of stay, complications, and revision rates. Their findings were confirmed by Wilson’s results of patients with >10 OME having worse outcomes, poorer wound healing, and extended admissions and ED utilization after discharge from arthroplasty procedures. Although the reasons are not yet understood, associations of higher complications, poor outcomes, and preoperative opioid use may suggest an underlying effect that these medications may have on patient recovery and rehabilitation. The increased OR between refill groups of postoperative complications may give evidence toward the idea that opioid medication may directly or indirectly contribute to the risk of postoperative complications. Clinical and basic science data strongly suggest a negative effect on bone density and healing with significantly increased rates of non-union in opioid users, and multiple studies have reported negative effects of opioids on bone remodeling.^28^ At this time, we are unable to differentiate whether complications increase the risk of opioid use or whether increased opioid use leads to an increased risk of postoperative events and complications. Although patients may benefit in acute pain relief from opioid medications, these medications may inhibit the healing process necessary in the post-surgical window.

There are inherent limitations to the interpretation of this study’s results. Relying on aggregated data sets, this study is limited by the coding accuracy for diagnoses and procedures in the MarketScan database. We are unable to control for fracture characteristics, including impaction and comminution that may affect outcomes. We were unable to identify and stratify the surgical techniques, quality of reduction, or specific hardware used. Our study additionally assumes that all prescriptions queried are directly related to the index surgical operation, which may not always be the case. Correlations between self-pay status and increased opioid consumption have been previously published^1^; however, limited by the MarketScan data, these patients are not included in our analysis. Although the comorbidity and factors identified in this study may influence a patient’s risk of prolonged opioid use, this study is not able to determine or recommend an optimal prescribing regimen for pain control after distal radius fracture fixation. Despite these limitations, the MarketScan database provides a large sample size and longitudinal data across the health care system. The size of our data set and the details available allow us an understanding of opioid prescription and consumption habits across the population. This study lacks many of the limitations and can control for many biases that are inherent in smaller or single institutional analyses.

## Conclusion

Many patient-specific factors for prolonged opioid consumption after distal radius fracture fixation can be identified before prescribing postoperative pain management. Hand surgeons historically prescribe a surplus of opioids to patients, and over-prescription may increase the risk of long-term use, complications, and poor outcomes. Depression, cardiovascular disease, diabetes, substance abuse, and medical and surgical complications all demonstrated an increased risk for the prolonged use of opioid medication after distal radius fracture fixation. Identifying these risk factors ahead of time allows the surgeons to educate patients and understand the risks in postoperative pain management. These results suggest that surgeons should take a more patient-specific approach for prescribing opioid medication after distal radius fracture fixation.
